# Modelling saliency attention to predict eye direction by topological structure and earth mover’s distance

**DOI:** 10.1371/journal.pone.0181543

**Published:** 2017-07-26

**Authors:** Longsheng Wei, Jian Peng, Wei Liu, Xinmei Wang, Feng Liu

**Affiliations:** 1 School of Automation, China University of Geosciences, Wuhan, China; 2 Hubei key Laboratory of Advanced Control and Intelligent Automation for Complex Systems, Wuhan, China; 3 Harvard Medical School, Boston, Massachusetts, United States of America; National University of Defense Technology College of Mechatronic Engineering and Automation, CHINA

## Abstract

A saliency attention model for predicting eye direction is proposed in this paper. This work is inspired by the success of the topological structure and Earth Mover’s Distance (EMD) approach. Firstly, we extract visual saliency features such as color contrast, intensity contrast, orientation, and texture. Then, we eliminate disconnected regions in the feature maps to keep topological structure. Secondly, we calculate center surround difference using across-scale EMD between different scales feature maps, rather than utilizing the Difference of Gaussian (DoG), which is used in many other saliency attention models. Thirdly, we across-scale fuse the feature maps in different scale and same feature. Lastly, we take advantage of competition function to calculate feature maps in same feature to form a saliency map, which is use to predict eye direction. Experimental results demonstrated the proposed model outperformed the state-of-the-art schemes in eye direction prediction community.

## 1. Introduction

When human observe the world, they attend to only a fragment of the total scene at any one moment. This phenomenon can be represented by visual attention and the conspicuity of each spatial region can be indicated by a saliency map. In human perception, attention is guided by this “bottom-up” salience as well as by “top-down” intentions of the viewer. The research of predicting eye direction focuses on identifying human gaze locations when they are observing natural scenes. In recent years, it has been receiving increasing interest that saliency attention was used to predict human eye direction [1].

Two basic theories about the primitives of visual perception are feature analysis theory and visual topology theory. The feature analysis theory assumes the process of visual perception is local-to-global. This theory considers that visual features are represented and processed with separate “feature maps” that are later integrated into a “saliency map” to orient visual attention [2]. However, the visual topology theory considers the visual perception to be a global-to-local process. It thinks that wholes are coded superior to their parts or separable properties. The core idea of topology theory is that perceptual structure should be understood under the perspective of perception and transformation of invariance in transformation [3].

The research of predicting eye direction contains three parts: feature extraction, contrast computation, and saliency map fusion. In the feature extraction part, Itti et al. proposed a set of biologically inspired visual features including color contrast, intensity contrast, and orientation [4]. Liu et al. proposed a set of novel features to detect salient object, this features including multi-scale contrast, center-surround histogram, and color spatial distribution [5]. Although this method has achieved good results, the biological principle of these features needs to be further studied. In contrast computation part, the most influential mechanism is called “center surround difference”. It utilized an operator of Difference of Gaussian (DoG) to calculate the contrast between the center location and surrounding region across multi-scale. In [6] and [7], they computed the contrast in image’s frequency domain, which manipulated the frequency spectrum to the salient locations. However, these algorithms are time consuming and difficult to achieve real-time application. The last part is to fuse various contrast features into a saliency map. Most models linearly combine different contrast features into a saliency map. Zhao et al. applied a least square algorithm to learn the optimal weight values based on a set of human eye tracking databases [8]. Because the weight values are quite different for different databases, it is hard to extend these weight values to other models. Furthermore, Harel et al. proposed a Graph-Based bottom-up visual saliency model [9], It consists of two steps: first forming activation maps on certain feature channels, and then normalizing them in a way which highlights conspicuity and admits combination with other maps. This model still uses Itti’s visual features and partial improves Itti’s model. Li et al. considered saliency detection as a frequency domain analysis problem and achieve this by employing the concept of nonsaliency [10]. Judd et al. adopted supervised learning algorithms to combine different feature maps [11]. It adopted supervised learning algorithms to combine different feature maps. Erdem et al. gave the visual saliency estimation by nonlinearly integrating features using region covariances [12]. Lu et al. proposed a visual saliency detection algorithm to explore the fusion of various saliency models in a manner of bootstrap learning [13]. Although these models have improved detection rate in their respective aspect, the effect is not very obvious.

**Fig 1:**
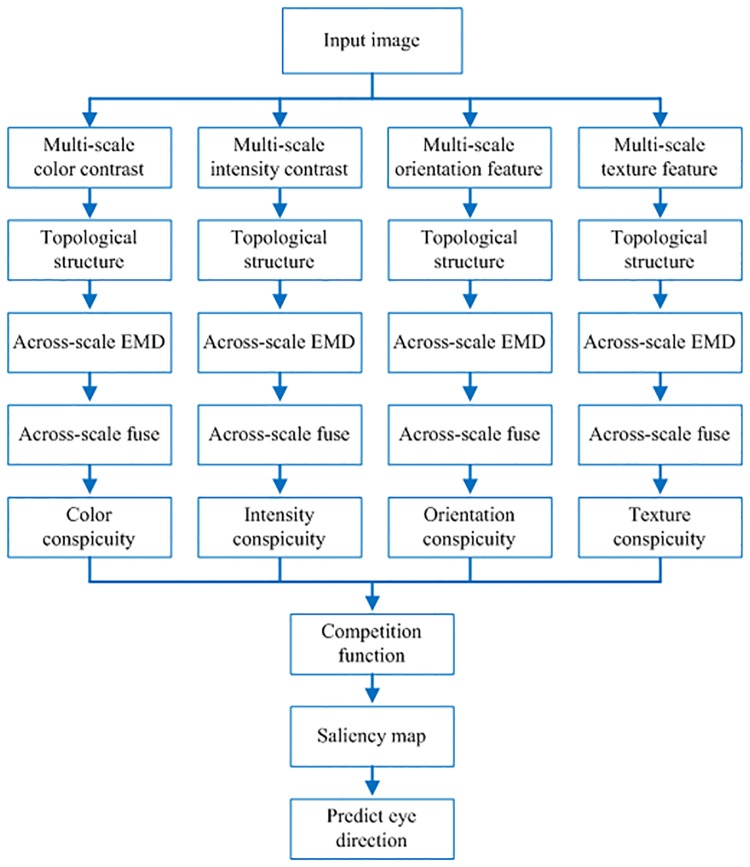
The frame work of our proposed saliency attention model.

In this paper, we aim to model a saliency attention to predict eye direction. We try to integrate feature analysis theory and visual topology theory into visual perception, and we employ Earth Mover’s Distance (EMD) to provide a more robust metric between the histograms of the center and surround regions. Our work is inspired by the success of the topological structure and EMD approach. Firstly, we extract visual saliency features such as color contrast, intensity contras, orientation, and texture. After that, we eliminate disconnected regions in feature maps to keep topological structure. Secondly, we calculate center surround difference using across-scale EMD between different scales feature maps. Lastly, we across-scale fuse the feature maps in same feature. Then, we take advantage of competition function to calculate feature maps in same feature to form a saliency map, which is use to predict eye direction. Our proposed saliency attention model is shown in Fig 1.

## 2. Method

In this part, we extract visual saliency features and eliminate disconnected regions in feature map to keep topological structure. Then we use across-scale EMD to calculate center surround difference. Finally, we across-scale fuse the feature maps to form a saliency map, which is use to estimation gaze direction.

In Itti et al. model [4], color, intensity and orientation are extracted for visual saliency features. Since human being is sensitive to image texture information, texture feature is added in this paper. Therefore, we extract ten visual features, including: two color contrasts (red/green contrast and blue/yellow contrast), two intensity contrasts (light-on-dark contrast and dark-on-light contrast), four orientation features (0°, 45°, 90°, 135°) and two texture features (original and extended LBP) from each input image [14].

### 2.1 Topological structure

Earlier researches have shown that human visual system is sensitive to the global properties of scene, so how to extract the global properties has become a fundamental problem in perceptual organization. A visual scene is usually separated into some different global wholes, like a foreground and a background, only dependent on the global properties. In [3], Chen presented those global properties could be described as the topological invariable properties, like connectivity. So when people speak of an object in the scene, they usually imply the object is connected.

Humans and all primates are sensitive to topological properties; in particular, the perception of physical connectivity is prior to the perception of geometrical properties [15]. The primitive visual perception is the invariants at different geometrical levels. According to descending order of stability and also from global to local, these properties include topological, projective, affine, and Euclidean geometrical invariants. Therefore, we select the topology properties as the global properties in this paper.

The topological properties mainly include connectedness, closedness and hole, while geometric properties such as angle, size, and parallelism do not belong to topological properties. We just consider the connectedness in this paper as it is the most representative property in the image processing.

We explore the connectivity for saliency visual attention. The essence of connectivity is a enclosure topological relationship in the different visual components. In mathematics, this topological relationship has invariant property under homeomorphisms. That is to say, it is a quite basic property of a visual scene, regardless of the shape or the scale of the visual content.

Formally, the image connectedness can be defined based on the connectivity of any two pixels in the image. On a binary map, an image patch is connected if there exists at least one path that joins any two given pixels in patch. Here, a path is a sequence of pixels in which any pair of consecutive pixels is adjacent. In this paper, we consider eight-adjacency. Therefore, a binary map can be separated into many absolute image patches according to the connectivity of the pixels.

**Fig 2:**
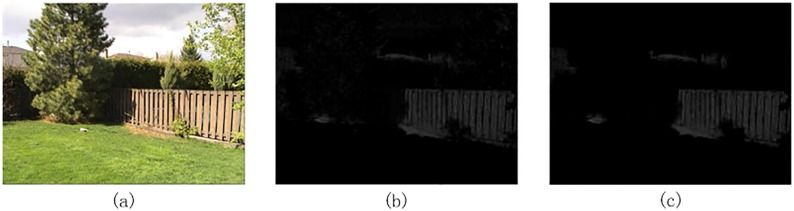
An illustration of the image topological structure. (a) Original image, (b) Feature map, (c) Topological connected feature map.

We can get the topological connected map in the following step. Firstly, we extract saliency feature maps from original image, and transform all the feature maps into binary maps. Secondly, we calculate the area of each isolated connected image patch, and then compute the ratio of every connected patch area to the total image area. Thirdly, we arrange all image patches in descending order, and select the top 10 patches or the patch with a ratio more than 10% as the topological connected binary map. Lastly, we obtain a topological connected feature map by masking the feature map with topological connected binary map. The process of obtaining the topology connected feature map is illustrated in Fig 2.

### 2.2 EMD

Previous visual attention models usually use center surround difference to represent the visual contrast of the feature maps, and they also use DoG to calculate the center and surround difference. The DoG filter is particularly performed by applying the Gaussian filter to a multi-scale image and then calculating their differences. However, point-to-point difference is sample and is hard to express the contrast information of image patches. EMD can afford a more robust metric about center surround by comparing their image patch histograms, so EMD is used to compute the difference in this part.

The classical EMD between two histograms is the lowest cost of transporting one histogram into another, in which the cost is usually defined as that the amount of weight multiplies the ground distance between two histograms. This formalization is easily generalized to two normalized histograms with the same number of bins [16].

Given two *n*-bin histograms $H^{1}=\{h_{i}^{1},i=1,2,3,\ldots ,n\}$ and $H^{2}=\{h_{j}^{2},j=1,2,3,\ldots ,n\}$. *H*^1^ is transformed into *H*^2^ by moving “mass” from $h_{i}^{1}$ to $h_{j}^{2}$ for every pair of (*i*, *j*), such that the difference of two histograms is minimized. Let another *n*-bin all-zero histogram be *T*, and we denote the flow *f*_*ij*_ as the amount, which is moved from the bin *i* in *H*^1^ to the bin *j* in *T*. Then we can define the EMD metric between *H*^1^ and *H*^2^ to be the minimum amount flow that is demanded to make the histogram of *T* to be identical with *H*^2^. Therefore, the EMD between *H*^1^ and *H*^2^ is expressed in mathematically as follow:

$$EMD(H^{1},H^{2})=\underset{\left\{f_{i,j},i,j=1,2,3,\ldots ,n\right\}}{\text{min}}\sum_{i=1}^{n}\sum_{j=1}^{n}f_{i,j}d_{i,j}$$ (1)

subject to

$$\sum_{j=1}^{n}f_{i,j}=h_{i}^{1},\;\sum_{i=1}^{n}f_{i,j}=h_{j}^{2},\;f_{i,j}\geq 0,\text{ and }i,j=1,2,3,\ldots ,n$$

where *d*_*i*, *j*_ denotes the ground distance between the *i*-th bin and the *j*-th bin. Let *d*_*i*, *j*_ = |*i* − *j*| to be the *L*^1^ distance in this section.

It is very complex to calculate the EMD between two histograms directly. Fortunately, with above choice, Levina et al. have proven that the EMD between normalized histograms equals to linear Wasserstein distance [17]. Under those conditions, the EMD can be written as:

$$EMD(H_{C},H_{S})=\sum_{i=1}^{n}\left|\sum_{j=1}^{i}H_{C}(j)-\sum_{j=1}^{i}H_{S}(j)\right|$$ (2)

where *H*_*C*_ and *H*_*S*_ denote the *n*-bin normalized center histogram and surround histogram, respectively.

### 2.3 Saliency map

The visual saliency map in this part is an expansion of a classical Itti’s model [4] since it not only uses EMD to calculate center and surround difference, but also adds texture feature, except for color, intensity and orientation features.

We construct an image pyramid with *m* scales to obtain different scales feature maps (color, intensity, orientation, and texture). For a given location (*x*, *y*), we select an adequate patch around this location, and we can find all corresponding patches in different scales. Let the small patch (coarser scale, *c* ∈ {1, 2, …, *m* − 2}) as center and big patch (fine scale, *s* = *c* + *δ*, *δ* ∈ {1, 2}) as the surround. Then we can calculate across-scale EMD maps for the center and surround patches in same feature. Because the bigger interval between layers in the pyramid, the smaller response value in the EMD map, while *δ* means the interlayer distance, we set 1/*δ* as weight value for cross-scale fusing EMD maps. Thus, we can obtain feature response conspicuity maps by combining all the across-scale EMD maps:

$$R_{f}(x,y)=\sum_{c}\sum_{s=c+\delta }\frac{1}{\delta }\left(EMD\left(H_{f,c}(x,y),H_{f,s}(x,y)\right)\right)$$ (3)

where *H*_*f*, *c*_ and *H*_*f*, *s*_ are the histograms for center and surround in terms of feature *f*.

We combine all the conspicuity maps into a saliency map. Let the competition algorithm of spatial feature be a function *ff*, then we use this function to combine different features into a visual saliency map [18].

$$S(x,y)=ff\left(\sum_{f}R_{f}(x,y)\right)$$ (4)

The saliency map shows the importance of each location in the visual scene. By “winner take all” mechanism and visual transfer method, the proposed model can achieve the goal of predicting eye direction.

## 3. Experiment

We use two benchmark eye tracking datasets MIT [11] and Toronto [19] to predict eye direction performance. They are the most widely used for evaluating eye direction prediction methods. MIT database consists of 15 participants on 1003 color images, including people portraits, indoor and outdoor daily life pictures. While Toronto database consists of 20 viewers on 120 color images, and a large portion of images do not contain particular regions of interest. For each image in both databases, the human density maps and the eye tracking fixations are given as the ground truth standard for evaluation.

**Fig 3:**
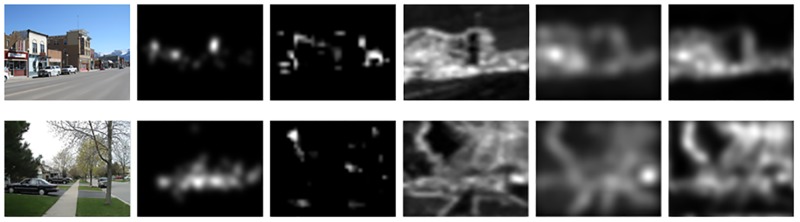
Two examples of four kinds saliency maps. The first column is input images, and the second column is the human eye density maps. The third to last column is the saliency maps, which are produced without topology and with DoG, without topology and with EMD, with topology and with DoG, with topology and with EMD, respectively.

In this paper, we introduce the topological structure and EMD into the calculation of the saliency map. In order to prove their effectiveness, we calculate four kinds saliency maps: without topology and with DoG (Itti model), without topology and with EMD, with topology and with DoG, with topology and with EMD (Proposed model). Fig 3 gives two examples of four kinds saliency maps.

A most widely used measure for saliency model evaluation is the metric of Area Under Curve (AUC). The curve is Receiver Operating Characteristic curve (ROC). For a given saliency map, a set of binary maps could be generated by varying threshold. Thus each binary map can represent a binary classification of the image pixels, in which positive pixels are eye fixations predictions. Then the False Positive Rate (FPR) and True Positive Rate (TPR) can be calculated based on a human eye ground truth fixation map. So it is easy to plot a ROC curve of FPR against TPR by varying the threshold to generate the binary images. After that, the AUC score is calculated as the area under ROC curve.

However, center bias has become a serious problem in visual attention. The image center regions are more possible to be saliency than the image perimeter regions [11], which poses a serious challenge for fair comparisons. To remove the effect of center bias in two datasets, a shuffled-AUC (sAUC) measure is proposed by [20], which has become a popular evaluation method in many recent researches [21]. We use the sAUC to compare the experiment results for different attention models in this paper.

We compare these four kinds saliency maps with human eye density maps in MIT and Toronto databases. The statistical results are shown in Table 1.

**Table 1:** Mean sAUC for four kinds saliency models in two databases.

	Without topology and with DoG	Without topology and with EMD	With topology and with DoG	With topology and with EMD
MIT	0.5628	0.6134	0.6347	0.6837
Toronto	0.5925	0.6581	0.6427	0.6960

From above experimental results, we easily can find that the values of the third column are larger than the values of the first column; the values of the fourth column are greater than the values of the second column. The results demonstrate that the saliency map with topology can better predict eye fixations than without topology. That is because human visual system is sensitive to the global properties of scene. By keeping topological structure, the saliency map eliminates the non-connected areas and highlights the global properties in the scene.

In Table 1, it is also found that the values of the second column are larger than the values of the first column; the values of the fourth column are greater than the values of the third column. The results indicate that the saliency map with EMD can better predict eye fixations than with DoG. The reason is that DoG uses the point-by-point difference, which is sample and is hard to express the contrast information of image patches, while EMD can afford a more robust metric about center surround by comparing their image patch histograms, which can keep global properties of the contrast.

**Fig 4:**
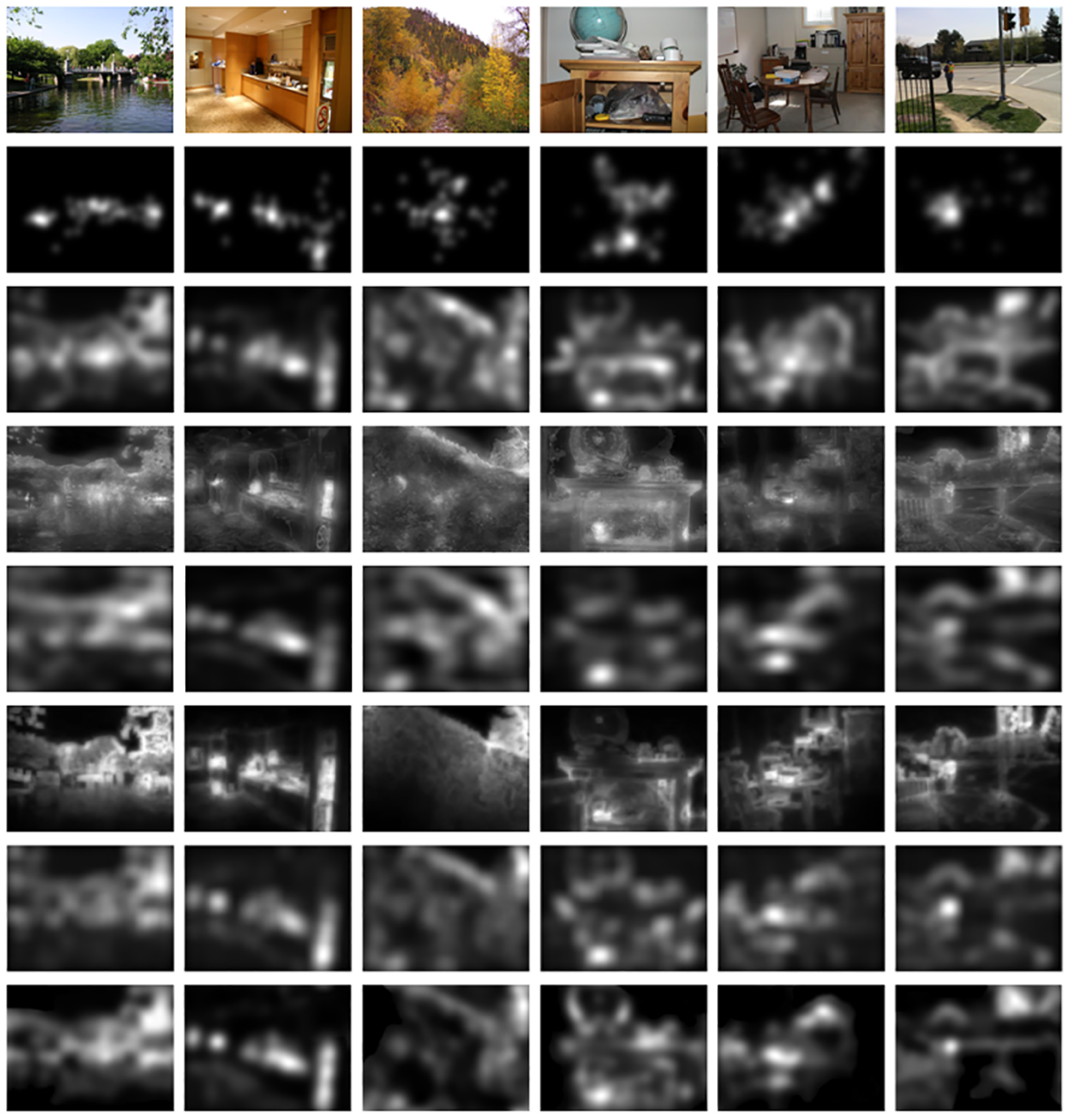
Some sample images and corresponding saliency maps produced by compared models. The first row is original images, and the second row is the human eye density maps. The third to last row are the saliency maps, which are produced by the model of Harel [9], Judd [11], HFT [10], Erdem [12], Lu [13], and our proposed, respectively. The left three images belong to the MIT database, and the right three images belong to the Toronto database.

Five state-of-the-art saliency models are evaluated for comparison, including Graph-based Harel’s model [9], machine learning Judd’s model [11], spectral domain HFT model [10], region covariances Erdem’s model [12], and bootstrapping Lu’s model [13]. Some sample images and saliency maps of compared models are shown in Fig 4.

**Fig 5:**
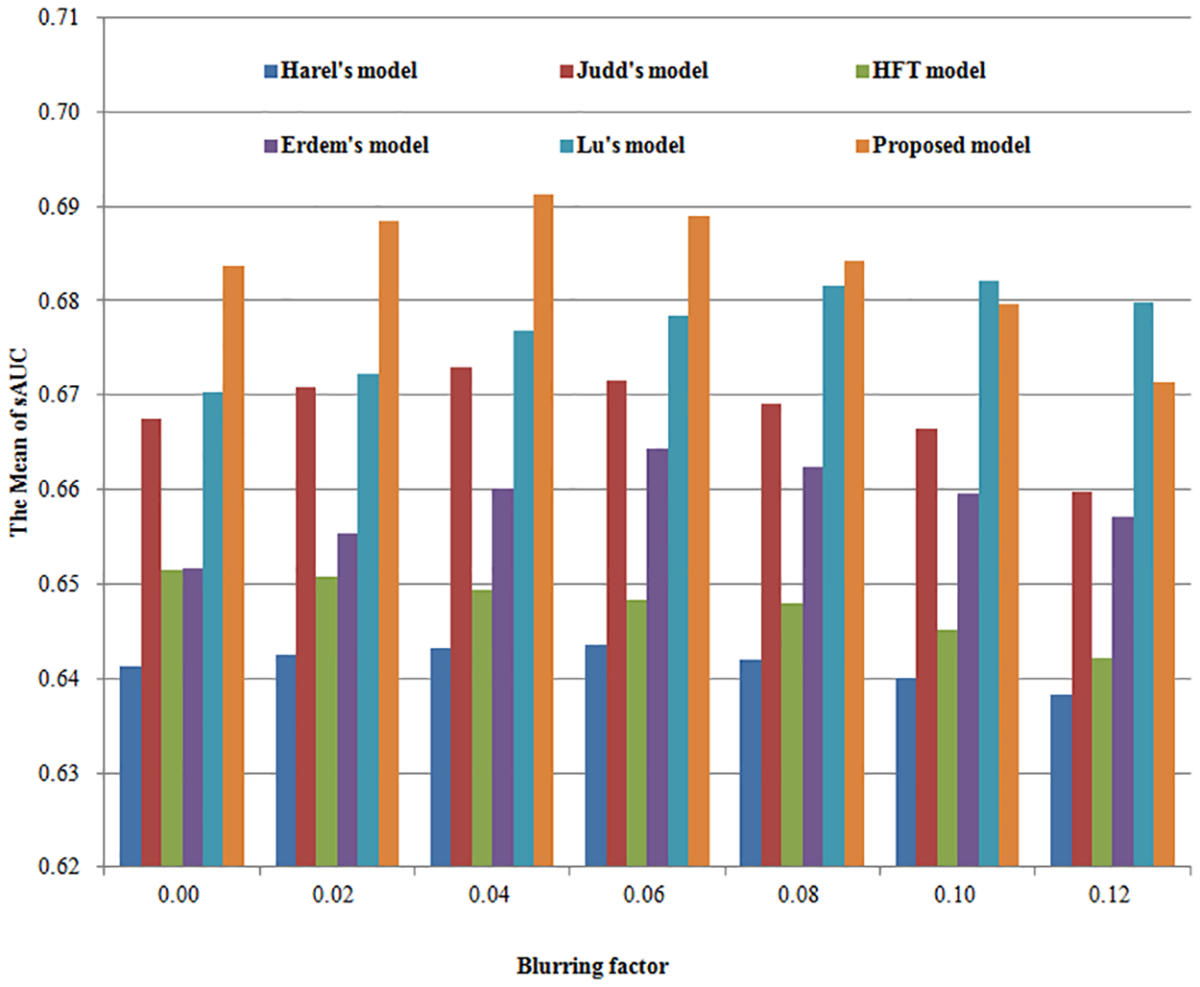
The comparison histograms of Mean sAUC for six different models in MIT database.

**Fig 6:**
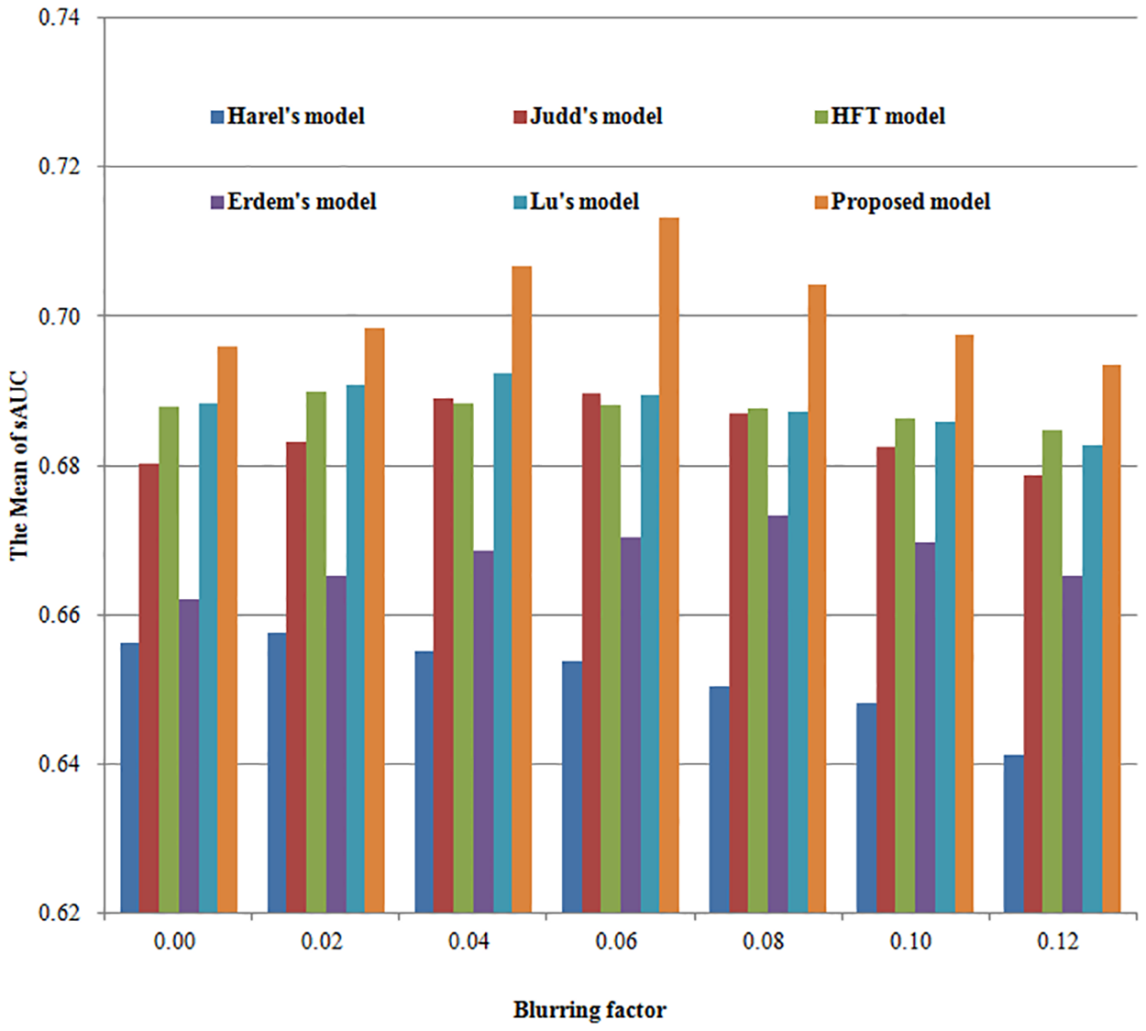
The comparison histograms of Mean sAUC for six different models in Toronto database.

Because we just want to compare the saliency maps without/with topological structure, with Dog, with EMD, we use the original saliency maps directly in Table 1. In order to compare the saliency maps of different models more accurately, we need to introduce blurring factors. Since blurring play a very important role in comparing different models, we post-process the saliency map by Gaussian blurring with standard deviation (STD). To achieve a better comparison, we exhaustively try all possible blurring factors for each compared model. We define the blurring factor STD as the percent of the image width, and we set the percent from 0 to 12% in experiment. The mean sAUC scores and the comparison histograms are presented in Table 2 and Fig 5 for MIT database. Table 3 and Fig 6 show the corresponding results for Toronto database.

**Table 2:** Mean sAUC for five attention models and our proposed model in MIT database.

Blurring factor	Harel’s model	Judd’s model	HFT model	Erdem’s model	Lu’s model	Proposed model
0.00	0.6412	0.6675	**0.6514**	0.6517	0.6704	0.6837
0.02	0.6425	0.6708	0.6507	0.6554	0.6722	0.6885
0.04	0.6432	**0.6729**	0.6494	**0.6601**	0.6769	**0.6913**
0.06	**0.6436**	0.6716	0.6483	**0.6643**	0.6785	0.6890
0.08	0.6420	0.6691	0.6479	0.6624	0.6816	0.6842
0.10	0.6401	0.6664	0.6452	0.6596	**0.6821**	0.6796
0.12	0.6383	0.6597	0.6421	0.6572	0.6798	0.6714

**Table 3:** Mean sAUC for five attention models and our proposed model in Toronto database.

Blurring factor	Harel’s model	Judd’s model	HFT model	Erdem’s model	Lu’s model	Proposed model
0.00	0.6562	0.6803	0.6879	0.6621	0.6884	0.6960
0.02	**0.6576**	0.6831	**0.6900**	0.6652	0.6909	0.6984
0.04	0.6551	0.6890	0.6884	0.6685	**0.6923**	0.7068
0.06	0.6537	**0.6897**	0.6881	**0.6703**	0.6895	**0.7132**
0.08	0.6504	0.6869	0.6876	**0.6734**	0.6872	0.7043
0.10	0.6481	0.6824	0.6863	0.6697	0.6859	0.6976
0.12	0.6413	0.6786	0.6847	0.6652	0.6827	0.6935

For each model, the optimal sAUC score is expressed in bold font. If we choose the optimal sAUC score as the experimental result of the model, we find that our proposed model outperforms the given five known models in both databases. The reason is that we not only introduced the topological structure into the initial feature processing, but also we use robust cross-scale EMD to calculate the central and surround difference.

## 4. Conclusion

We have proposed a saliency attention model based on topological structure and EMD to predict eye direction in this paper. Visual saliency features are extracted from input visual scene, and the disconnected regions are eliminated in feature maps to keep topological structure. Then, across-scale EMD is used to calculate the center and surround difference between different scales feature maps. Thus the feature maps are across-scale fused in same feature. Lastly, a spatial competition function is used to combine different feature maps into a visual saliency map. Experimental results demonstrated that the comparison indexes of the proposed models were better than the indexes of given models.

Two key contributions are made in the paper, one is that we introduce the topological structure into the processing of feature maps, and the other is that we use EMD to calculate the center and surround difference instead of DoG filter. In future work, we will add depth information in feature extraction, and extend the model for saliency detection in videos.
